# Aerosolized antibiotics for ventilator-associated pneumonia: a pairwise and Bayesian network meta-analysis

**DOI:** 10.1186/s13054-018-2106-x

**Published:** 2018-11-15

**Authors:** Feng Xu, Lu-Lu He, Luan-Qing Che, Wen Li, Song-Min Ying, Zhi-Hua Chen, Hua-Hao Shen

**Affiliations:** 1 Key Laboratory of Respiratory Disease of Zhejiang Province, Department of Respiratory and Critical Care Medicine, The Second Affiliated Hospital, Zhejiang University School of Medicine, Hangzhou, China; 2 State Key Laboratory of Respiratory Disease, Guangzhou, China

**Keywords:** Aerosolized antibiotics, Ventilator-associated pneumonia, Multidrug-resistant pathogens, Meta-analysis, Network meta-analysis

## Abstract

**Background:**

Aerosolized antibiotics have been proposed as a novel and promising treatment option for the treatment of ventilator-associated pneumonia (VAP). However, the optimum aerosolized antibiotics for VAP remain uncertain.

**Methods:**

We included studies from two systematic reviews and searched PubMed, EMBASE, and Cochrane databases for other studies. Eligible studies included randomized controlled trials and observational studies. Extracted data were analyzed by pairwise and network meta-analysis.

**Results:**

Eight observational and eight randomized studies were identified for this analysis. By pairwise meta-analysis using intravenous antibiotics as the reference, patients treated with aerosolized antibiotics were associated with significantly higher rates of clinical recovery (risk ratio (RR) 1.21, 95% confidence interval (CI) 1.09–1.34; *P* = 0.001) and microbiological eradication (RR 1.42, 95% CI 1.22–1.650; *P* < 0.0001). There were no significant differences in the risks of mortality (RR 0.88, 95% CI 0.74–1.04; *P* = 0.127) or nephrotoxicity (RR 1.00, 95% CI 0.72–1.39; *P* = 0.995). Using network meta-analysis, clinical recovery benefits were seen only with aerosolized tobramycin and colistin (especially tobramycin), and microbiological eradication benefits were seen only with colistin. Aerosolized tobramycin was also associated with significantly lower mortality when compared with aerosolized amikacin and colistin and intravenous antibiotics. The assessment of rank probabilities indicated aerosolized tobramycin presented the greatest likelihood of having benefits for clinical recovery and mortality, and aerosolized colistin presented the best benefits for microbiological eradication.

**Conclusions:**

Aerosolized antibiotics appear to be a useful treatment for VAP with respect to clinical recovery and microbiological eradication, and do not increase mortality or nephrotoxicity risks. Our network meta-analysis in patients with VAP suggests that clinical recovery benefits are associated with aerosolized tobramycin and colistin (especially tobramycin), microbiological eradication with aerosolized colistin, and survival with aerosolized tobramycin, mostly based on observational studies. Due to the low levels of evidence, definitive recommendations cannot be made before additional, large randomized studies are carried out.

**Electronic supplementary material:**

The online version of this article (10.1186/s13054-018-2106-x) contains supplementary material, which is available to authorized users.

## Background

Ventilator-associated pneumonia (VAP), one form of hospital-associated pneumonia (HAP), is defined as pneumonia developing in a mechanically ventilated patient ≥ 48 h after tracheal intubation. When it occurs, VAP has been recognized as the leading cause of mortality among patients with nosocomial infections, and VAP patients are hospitalized on average for an additional 4–13 days with excess hospital costs [1]. Effective antimicrobial therapy requires adequate drug concentrations at the infection site. However, intravenous therapy has altered the pharmacokinetics and poor lung tissue penetration of many antimicrobial agents [2]. Thus, aerosolized antibiotic therapy directly targets airway and lung parenchyma resulting in high local concentrations and potentially higher clinical responses. Moreover, aerosolized antibiotic therapy, which directly treats lung infections, can be used to decrease systemic antibiotic doses to minimize antibiotic-associated toxicities [3].

Two previous meta-analyses reported that aerosolized antibiotic therapy might be beneficial in the treatment of VAP [4, 5]. However, all of these studies were traditional pairwise meta-analyses comparing aerosolized antibiotics with intravenous antibiotics, and none made comparisons between the aerosolized antibiotics. A network meta-analysis (NMA), also known as mixed treatment comparison or multiple treatment comparison, is a method for concurrent comparison of multiple treatments in a single meta-analysis [6].

In the present study, our objective was to update the evidence to systematically evaluate the effect of aerosolized antibiotics in VAP patients on clinical recovery, microbiological eradication, mortality, and nephrotoxicity; we also used an NMA approach which enabled us to assess three aerosolized antibiotics by indirect comparison to determine their efficacy.

## Methods

### Search strategy

We included the studies from two systematic reviews [4, 5]. In addition, we also searched the PubMed, EMBASE, and Cochrane databases from inception to September 2017 to identify potentially relevant studies. Search terms included several parameters: 1) ventilator associated pneumonia OR VAP OR respiratory infection OR respiratory tract OR hospital acquired pneumonia OR nosocomial pneumonia; 2) aerolised OR aerosolized OR inhaled OR nebulised OR nebulized. We also evaluated the reference lists of the relevant clinical trials to identify additional studies.

Studies were included if they met several criteria: 1) patients in a study population that were mechanically ventilated and diagnosed with VAP; 2) intervention which included the use of inhaled antibiotics for treatment of VAP compared with intravenous antibiotics; and 3) at least one of the following outcomes was reported: clinical recovery, microbiological eradication, mortality, and nephrotoxicity.

### Study quality assessment

The Newcastle-Ottawa scale (NOS) for observational studies was used to assess the quality of the included studies. The NOS statement was judged on three broad perspectives (selection, comparability, and outcome) consisting of eight items. The qualities of randomized clinical trials were assessed using the risk of bias tool recommended by the Cochrane Collaboration. We assigned a value of high, unclear, or low to several parameters: 1) random sequence generation; 2) allocation concealment; 3) blinding of participants and personnel; 4) blinding of outcome assessment; 5) incomplete outcome data; 6) selective reporting; and/or 7) other bias.

### Statistical analysis

We used the STATA program (Stata Corporation, College Station, Texas) for pairwise meta-analysis. The differences between the two groups were calculated as the relative risk (RR) and 95% confidence interval (CI) for dichotomous outcomes. Heterogeneity was assessed by the Cochran *Q* statistic and the *I*^2^ statistic. A *P* value ≤ 0.10 together with an *I*^2^ value ≥ 50% indicates significant heterogeneity. *I*^2^ values ≤ 50% represented acceptable between-study heterogeneity, and the fixed-effects model was selected. Otherwise, the random-effects model was selected [7, 8]. Publication bias was determined using the funnel plot and assessed by Egger’s test. We performed the NMA within a Bayesian framework using JAGS (version 4.2.0), R software (version 3.4.4), and the rjags and gemtc packages. The probability that each aerosolized antibiotic therapy was the best among the given treatments was determined by evaluating the rank probabilities. A higher probability of achieving rank 1 indicated a higher probability of being the best. We used these results for our interpretation.

## Results

### Selection and characteristics of the studies

**Fig. 1:**
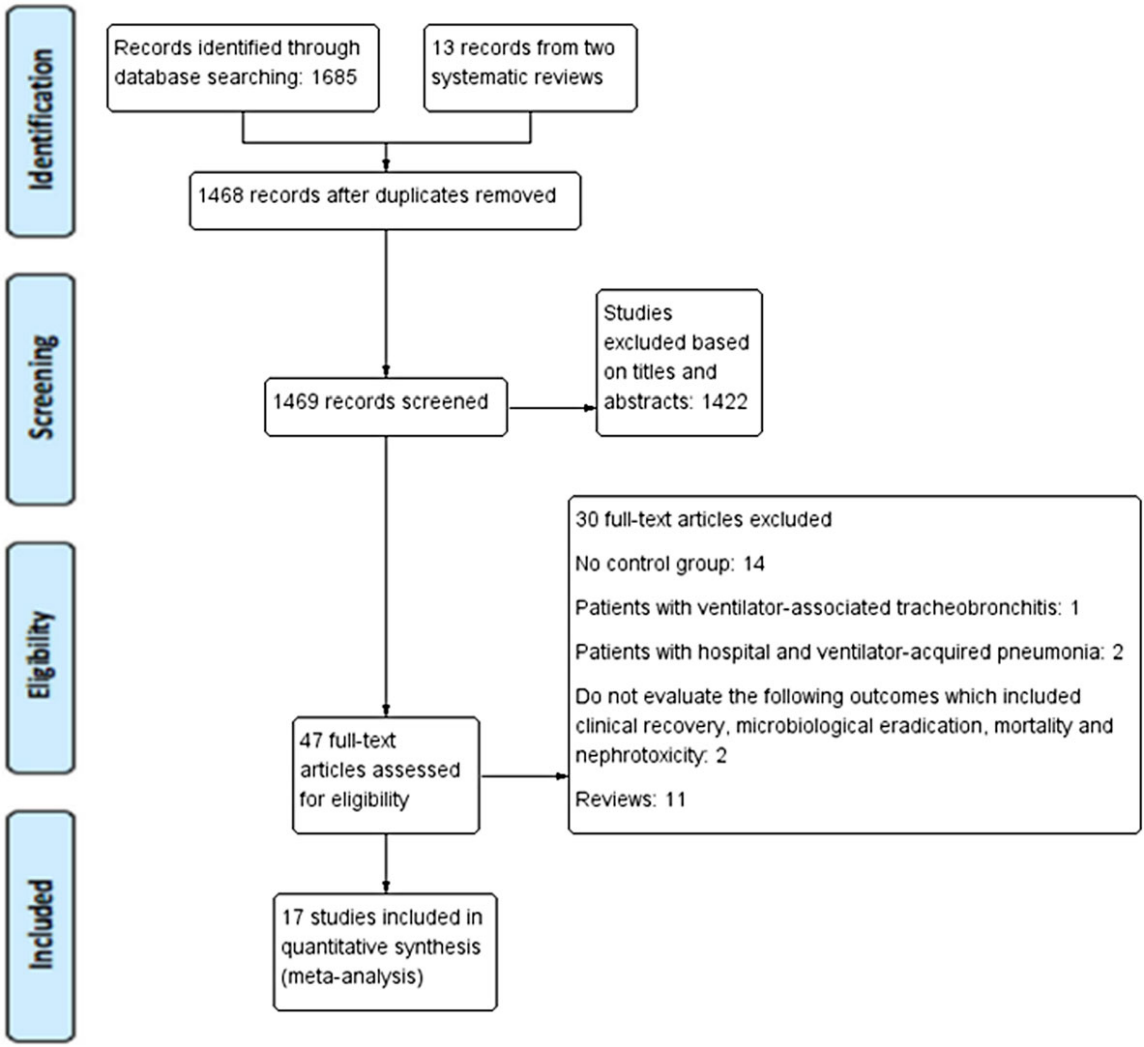
Study flow diagram

We identified 13 studies through the reanalysis of the two systematic reviews, and four studies through the PubMed, EMBASE, and Cochrane search. These trials were published between 2000 and 2017. Accordingly, the current meta-analysis included 17 studies [9–25]. The detailed steps of the study selection process are shown in Fig. 1.

Table 1 shows the major characteristics of the included trials. There were nine observational studies and eight randomized clinical trials. Fifteen studies presented the outcome of clinical recovery [9–21, 23, 24], and 10 presented the outcome of microbiological eradication [9, 10, 12–15, 19–22]. Mortality was reported in 14 trials [10, 11, 13–17, 19–25], and nephrotoxicity was reported in six trials [10, 13, 15, 18, 19, 21]. For the network meta-analysis, we excluded studies that used several inhaled antibiotics [9, 22, 25]. Three studies investigated the effect of inhaled amikacin [20, 21, 24], three studies concerned inhaled tobramycin [16–18], and eight studies concerned colistin [10–15, 19, 23].

**Table 1:** Characteristics of studies included in the meta-analysis

Study, year	Country	Inclusion population	No. of patients	Disease severity		Respiratory comorbidities		Administration strategy	Antibiotic given (dose)		Device for drug delivery	Main outcomes	Quality assessment/Cochrane risk of bias^a^
			(AS/IV)	AS	IV	AS	IV		AS	IV			
Observational studies													
Ghannam, 2009 [9]	USA	Gram-negative bacteria VAP	16/16	CPIS score: 7 ± 2.9	CPIS score: 6 ± 1.8	COPD: 3	COPD: 6	Substitution strategy	Colistin (100 mg every 8 h), tobramycin (300 mg b.i.d), gentamicin (100 mg t.i.d), and amikacin (100 mg t.i.d. or 300 mg b.i.d)	Amikacin (100 mg per 3 ml), gentamicin (40 mg per ml), and colistin (75 mg per 4 ml)	Jet nebulizer	Clinical recovery, microbiological eradication	9
Kofteridis, 2010 [10]	Greece	MDR VAP due to Gram-negative bacteria	43/43	APACHE II score: 16.95 ± 6.59	APACHE II score: 17.74 ± 7.61	COPD: 12	COPD: 7	Adjunctive strategy	Colistin (2 million IU b.i.d)	Colistin (3 million IU t.i.d)	Not described	Clinical recovery, microbiological eradication, all-cause mortality, nephrotoxicity	9
Korbila, 2010 [11]	Greece	Microbiologically documented VAP	78/43	APACHE II score: 17.4 ± 6	APACHE II score: 19.2 ± 7	Pulmonary: 17	Pulmonary: 9	Adjunctive strategy	Colistin (1 million IU)	Colistin (6.4 ± 2.3 million)	Ultrasonic nebulizer	Clinical recovery, mortality	9
Pérez-Pedrero, 2011 [12]	Spain	VAP due to multi-resistant *Acinetobacter baumannii*	36/18	APACHE II score: 11.2 ± 4.3	APACHE II score: 12.8 ± 5.7	Not described	Not described	Adjunctive strategy	Colistin (1 million every 8 h, 0.5 million every 8 h, or 1 million b.i.d)	Colistin (1 million every 8 h, 0.5 million every 8 h, or 1 million b.i.d)	Not described	Clinical recovery, microbiological eradication	8
Kalin, 2012 [13]	Turkey	VAP due to multi-resistant *A. baumannii*	15/29	APACHE II score: 22	APACHE II score: 22	COPD: 4	COPD: 6	Adjunctive strategy	Colistin (150 mg b.i.d)	Colistin (2.5 mg/kg b.i.d or every 6 h)	Not described	Clinical recovery, microbiological eradication, all-cause mortality, nephrotoxicity	9
Arnold, 2012 [25]	USA	*Pseudomonas aeruginosa* and *A. baumannii* VAP	19/74	APACHE II score: 17.5 ± 5.3	APACHE II score: 21.4 ± 5.7	Pulmonary: 22	Pulmonary: 6	Adjunctive strategy	Colistin (150 mg b.i.d) or tobramycin (300 mg b.i.d)	Standard IV antibiotics	Not described	All-cause mortality	8
Doshi, 2013 [14]	USA	MDR VAP due to Gram-negative bacteria	44/51	APACHE II score: 22.4 ± 7.1	APACHE II score: 24 ± 6.9	Not described	Not described	Adjunctive strategy	Colistin (75–150 mg b.i.d)	Colistin (2.5 mg/kg b.i.d)	Jet or vibrating mesh nebulizer	Clinical recovery, microbiological eradication, hospital mortality	7
Tumbarello, 2013 [15]	Italy	Patients with VAP caused by *Acinetobacter*, *Pseudomonas*, or *Klebsiella*	104/104	CPIS: 7.8 ± 1.2	CPIS: 7.9 ± 1.3	COPD: 21	COPD: 28	Adjunctive strategy	Colistin (1 million IU t.i.d)	Colistin (0.1 IU/kg every 8 to 12 h)	Jet or ultrasonic nebulizer	Clinical recovery, microbiological eradication, all-cause mortality, nephrotoxicity	9
Migiyama, 2017 [16]	Japan	ARDS patients with VAP caused by *P. aeruginosa*	22/22	APACHE II score: 26.2 ± 6.6	APACHE II score: 24.5 ± 7.0	Pulmonary: 6	Pulmonary: 5	Adjunctive strategy	Tobramycin (240 mg)	Tobramycin	Ultrasonic nebulizer	Clinical recovery, ICU mortality	9
Le Conte, 2000 [17]	France	Intubated and mechanically ventilated patients with nosocomial pneumonia	21/17	N/A	N/A	Not described	Not described	Adjunctive strategy	Tobramycin (6 mg/kg/day)	Betalactam and tobramycin	Pneumatic nebulizer	Clinical recovery, mortality	High
Hallal, 2007 [18]	USA	VAP caused by *Pseudomonas* or *Acinetobacter*	5/5	APACHE II score: 17 ± 1.26	APACHE II score: 15 ± 3.3	Not described	Not described	Substitution strategy	Tobramycin (300 mg b.i.d)	Betalactam and tobramycin	Jet nebulizer	Clinical recovery, nephrotoxicity	High
Rattanaumpawan, 2010 [19]	Thailand	Gram-negative bacteria VAP	51/49	APACHE II score: 19.1 ± 5.8	APACHE II score: 18.5 ± 4.7	Not described	Not described	Adjunctive strategy	Colistin (75 mg b.i.d)	Standard intravenous antibiotics	Jet or ultrasonic nebulizer	Clinical recovery, microbiological eradication, 28-day mortality, nephrotoxicity	High
Lu, 2011 [20]	France	VAP caused by *Pseudomonas*	20/20	CPIS score: 8 (7–8)	CPIS score: 9 (8–9)	COPD: 3	COPD: 4	Substitution strategy	Amikacin (25 mg/kg/day)	Amikacin (15 mg/kg/day) and ceftazidime (90 mg/kg/3 h)	Vibrating nebulizer	Clinical recovery, microbiological eradication, 28-day mortality	High
Niederman, 2012 [21]	France/Spain/USA	Mechanically ventilated patients with Gram-negative pneumonia	46/22	CPIS score: 6.8 (1.2)	CPIS score: 7 (1.2)	Not described	Not described	Adjunctive strategy	Amikacin (400 mg b.i.d or 400 mg/day)	Standard intravenous antibiotics	Vibrating mesh nebulizer	Clinical recovery, microbiological eradication, all-cause mortality, nephrotoxicity	Low
Palmer, 2014 [22]	USA	Patients with high risk for MDR organisms in the respiratory tract	24/18	APACHE II score: 20.96 ± 5.8	APACHE II score: 14.4 ± 5.5	COPD: 3	COPD: 2	Adjunctive strategy	Vancomycin (120 mg t.i.d), gentamicin sulfate (80 mg t.i.d), or amikacin (400 mg t.i.d)	Standard IV antibiotics	Jet nebulizer	Microbiological eradication, all-cause mortality	High
Abdellatif, 2016 [23]	Tunisia	Gram-negative bacteria VAP	73/76	SOFA score: 7.03 ± 3.8	SOFA score: 6.5 ± 4.1	Pulmonary: 32	Pulmonary: 29	Adjunctive strategy	Colistin (4 million IU t.i.d)	Imipenem (1 g t.i.d)	Ultrasonic vibrating plate nebulizer	Clinical recovery, 28-day mortality	Low
Kollef, 2017 [24]	USA	Gram-negative bacteria VAP	71/72	APACHE II score: 18.5 ± 5.6	APACHE II score: 18.4 ± 5.9	Not described	Not described	Adjunctive strategy	Amikacin (300 mg) and fosfomycin (120 mg)	Meropenem or imipenem	Vibrating plate electronic nebulizer	Clinical recovery, 28-day mortality	Low

*APACHE* Acute Physiology and Chronic Health Evaluation, *AS* aerosolized, *b.i.d.* twice daily, *COPD* chronic obstructive pulmonary disease, *CPIS* Clinical Pulmonary Infection Score, *ICU* intensive care unit, *IU* international units, *IV* intravenous, *MDR* multidrug resistant, *N/A* indicates not available, *SOFA* Sequential Organ Failure Assessment, , *t.i.d.* three times daily, *VAP* ventilator-associated pneumonia

^a^Quality assessment is shown for observational studies, and Cochrane risk of bias is shown for randomized controlled trials

### Pairwise meta-analysis

The overall pooled RR of clinical recovery, microbiological eradication, mortality, and nephrotoxicity are shown in Additional file 1: Figure S1. Additional file 1: Figure S1A shows the estimates of clinical recovery rates from fifteen studies. Aerosolized antibiotics were associated with significantly higher rates of clinical recovery (RR 1.21, 95% CI 1.09–1.34; *P* = 0.001). There was no statistical heterogeneity in the fixed effect model (*I*^2^ = 36.2%).

Ten trials reported microbiological eradication as an outcome, and the pooled results indicated that aerosolized antibiotics also had a beneficial effect (RR 1.42, 95% CI 1.22–1.65; *P* < 0.0001), despite significant heterogeneity among the studies (*I*^2^ = 68.5%) (Additional file 1: Figure S1B).

Mortality at the longest follow-up was available in 14 studies; aerosolized antibiotics had similar effects to control groups (RR 0.88, 95% CI 0.74–1.04; *P* = 0.127) with no evidence of statistical heterogeneity (*I*^2^ = 23.6%) (Additional file 1: Figure S1C).

Six studies presented data regarding complications on nephrotoxicity; aerosolized antibiotics were not associated with an increased risk of nephrotoxicity (RR 1.00, 95% CI 0.72–1.39; *P* = 0.995) with no evidence of statistical heterogeneity (*I*^2^ = 19.4%) (Additional file 1: Figure S1D).

### Publication bias

We detected no evidence of publication bias after assessing funnel plots (Additional file 2: Figure S2) and Egger’s test (clinical recovery *P* = 0.553, microbiological eradication *P* = 0.156, mortality *P* = 0.869 and nephrotoxicity *P* = 0.223). The data suggested that there was no evidence of publication bias for clinical recovery, microbiological eradication, mortality and/or nephrotoxicity.

### Sensitivity analyses

Sensitivity analyses were carried out to determine the influence of each study on the pooled RR, and the statistical findings were not materially altered by the elimination of any study (Additional file 3: Figure S3).

### Bayesian network meta-analysis

**Fig. 2:**
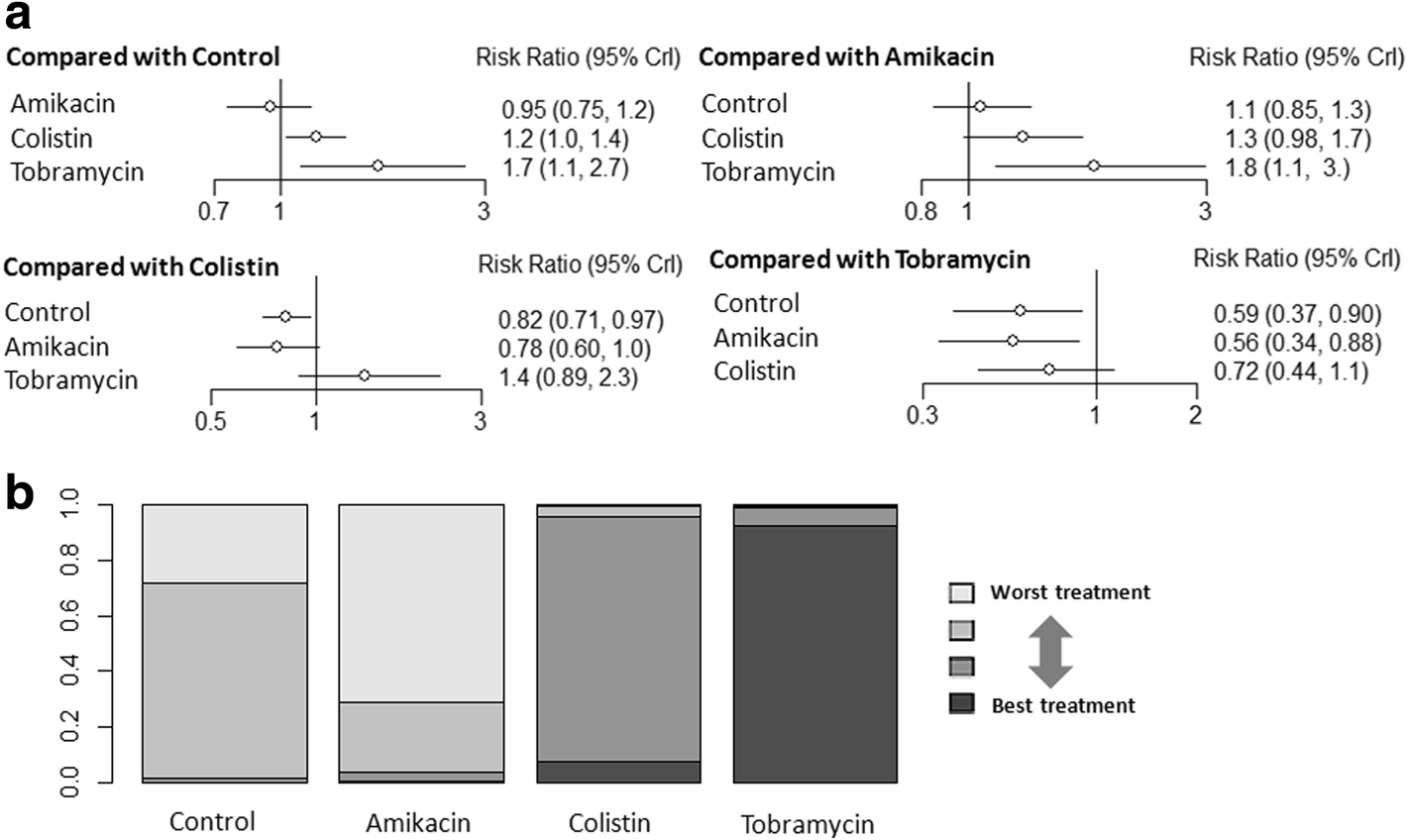
**a** Network estimates among aerosolized antibiotics for clinical recovery. **b** Rank probabilities among aerosolized antibiotics for clinical recovery based on the network meta-analysis. The number of patients in each antibiotic arm: control, 476; amikacin, 135; colistin, 372; and tobramycin, 48. CI confidence interval

There were no trials comparing outcomes among the aerosolized antibiotics, and all trials had an intravenous antibiotics arm. Thus, we pooled direct comparisons to obtain indirect comparisons by comparing aerosolized tobramycin versus aerosolized colistin, aerosolized tobramycin versus aerosolized amikacin, and aerosolized colistin versus aerosolized amikacin. In individual comparisons for clinical recovery using intravenous antibiotics as the reference, aerosolized tobramycin and colistin were more likely to increase the rate of clinical recovery (NMA: RR 1.7, 95% CI 1.1–2.7; and RR 1.2, 95% CI 1.0–1.4, respectively; Fig. 2a). Aerosolized tobramycin was associated with a significantly higher rate of clinical recovery compared with aerosolized amikacin (NMA: RR 1.8, 95% CI 1.1–3.0; Fig. 2a). There were no significant differences among the other comparisons of the network meta-analysis for clinical recovery (Fig. 2). The assessment of rank probabilities indicated that tobramycin (92.3% probability) presented the greatest likelihood of improving efficacy among the evaluated aerosolized antibiotics for treating VAP, followed by colistin (SUCRA 7.4% probability) and then amikacin (0.2% probability) (Fig. 2b). We conducted subgroup analyses according to geography, type of inhaled drug delivery system, VAP with or without multidrug resistance (MDR), type of studies, and administration strategy (Table 2). Results of our subgroup analyses from rank probabilities suggested that tobramycin displayed the best benefit for clinical recovery, followed by colistin, and then amikacin.

**Table 2:** Results of subgroup analysis

Outcome	Subgroup	Relative risk (95% confidence interval) and rank probabilities					
		Tobramycin vs control; tobramycin (rank probability)	Colistin vs control; colistin (rank probability)	Amikacin vs control; amikacin (rank probability)	Tobramycin vs colistin	Tobramycin vs amikacin	Colistin vs amikacin
Clinical recovery	Geography						
	USA	2.37 (1.7–5.62); tobramycin 97.3%	1.3 (0.68–2.7); colistin 2.2%	0.68 (0.27–1.7); amikacin 0.2%	1.83 (1.2–4.67)	3.4 (2.2–8.89)	2 (0.65–6.3)
	Europe	1.52 (0.52–6.6); tobramycin 65.4%	1.2 (0.86–1.5); colistin 28.7%	0.98 (0.67–1.5); amikacin 4.6%	1.3 (0.42–5.7)	1.6 (0.5–7.1)	1.3 (0.72–1.9)
	Asia	1.6 (0.74–3.3); tobramycin 82.3%	0.95 (0.48–1.9); colistin 5.6%	–	1.6 (0.60–4.5)	–	–
	Type of inhaled drug delivery system						
	Jet nebulizer	2.1 (1.5–5.34); tobramycin 98.6%	1.1 (0.71–1.8); colistin 1.2%	–	2.53 (1.2–6.7)	–	–
	Ultrasonic nebulizer	1.5 (0.85–3); tobramycin 78.8%	1.2 (0.95–1.6); colistin 20.5%	–	1.3 (0.67–2.6)	–	–
	Vibrating nebulizer	–	1.3 (0.93–2); colistin 91.9%	0.95 (0.72–1.2); amikacin 4.2%	–	–	1.4 (0.91–2.3)
	VAP with or without MDR						
	VAP with MDR	–	1.2 (0.70–1.7); colistin 66.4%	0.97 (0.40–2.5); amikacin 24.8%	–	–	1.2 (0.40–3.1)
	VAP without MDR	1.7 (1.1–3.2); tobramycin 88.5%	1.2 (0.83–1.7); colistin 9%	0.68 (0.27–1.6); amikacin 2.1%	1.5 (0.8–2.9)	2.6 (0.94–7.8)	1.8 (0.68–4.8)
	Type of studies						
	Randomized controlled trials	2.2 (0.95–8); tobramycin 92.9%	1.1 (0.71–1.8); colistin 5.6%	0.94 (0.65–1.3); amikacin 0.8%	2.5 (0.93–9.9)	2.4 (0.96–8.7)	1.2 (0.69–2.1)
	High risk of bias	2.2 (0.91–7.7); tobramycin 87.5%	0.94 (0.43–2); colistin 5.6%	0.98 (0.47–2.1); amikacin 5.7%	2.4 (0.74–10)	2.3 (0.73–9.5)	0.96 (0.33–2.8)
	Low risk of bias	–	1.4 (0.73–2.8); colistin 82.1%	0.92 (0.59–1.3); amikacin 7.7%	–	–	1.5 (0.72–3.5)
	Observational studies	1.6 (0.78–3.3); tobramycin 75.9%	1.2 (0.88–1.6); colistin 22.9%	–	1.3 (0.63–2.9)	–	–
	NOS = 9	1.6 (0.48–5.1); tobramycin 70.8%	0.96 (0.51–1.81); colistin 23.7%	–	1.3 (0.40–5.5)	–	–
	NOS < 9	–	1.2 (0.84–1.9); colistin 87.6%	–	–	–	–
	Administration strategy						
	Adjunctive strategy	1.6 (0.94–2.70); tobramycin 81.4%	1.2 (1.0–1.4); colistin 16.8%	0.93 (0.64–1.3); amikacin 1.4%	1.3 (0.76–2.3)	1.7 (0.92–3.2)	1.3 (0.87–2)
	Substitution strategy	1.57 (0.46–5.34); tobramycin 98.9%	–	0.96 (0.76–1.21); amikacin 0.7%	–	1.64 (0.47–5.69)	–
Mortality	Geography						
	USA	–	1.4 (0.76–2.6); colistin 3.6%	0.66 (0.30–1.4); amikacin 83.9%	–	–	2.1 (0.79–5.8)
	Europe	0.31 (0.039–1.6); tobramycin 85.7%	1.2 (0.40–4.1); colistin 0.1%	0.87 (0.57–1.3); amikacin 7.8%	0.36 (0.044–5.0)	0.25 (0.022–1.8)	0.72 (0.20–2.3)
	Asia	0.33 (0.074–1.4); tobramycin 86.9%	0.92 (0.26–3.3); colistin 10.6%	–	0.36 (0.051–2.4)	–	–
	Type of inhaled drug delivery system						
	Jet nebulizer	–	0.88 (0.63–1.2); colistin 81.6%	–	–	–	–
	Ultrasonic nebulizer	0.33 (0.11–0.87); tobramycin 97.2%	0.92 (0.64–1.4); colistin 1.7%	–	0.36 (0.11–1.0)	–	–
	Vibrating nebulizer	–	1.3 (0.70–2.4); colistin 20%	1.3 (0.68–2.6); amikacin 14.8%	–	–	1.0 (0.40–2.4)
	VAP with or without MDR						
	VAP with MDR	–	0.81 (0.55–1.2); colistin 70.9%	1.3 (0.39–6.3); amikacin 22.3%	–	–	0.61 (0.12–2.1)
	VAP without MDR	0.34 (0.13–0.78); tobramycin 71.7%	0.91 (0.54–1.5); colistin 7.4%	0.7 (0.3–1.7); amikacin 26.7%	0.37 (0.12–1)	0.48 (0.13–1.6)	1.3 (0.47–3.5)
	Type of studies						
	Randomized controlled trials	0.31 (0.039–1.6); tobramycin 80.9%	0.95 (0.45–2); colistin 6.6%	0.83 (0.41–1.8); amikacin 11.3%	0.32 (0.036–2)	1.1 (0.38–3.1)	0.37 (0.042–2.3)
	High risk of bias	0.31 (0.039–1.8); tobramycin 73.5%	0.92 (0.32–2.7); colistin 8%	1.1 (0.11–10); amikacin 16.3%	0.33 (0.033–2.7)	0.28 (0.013–5.2)	0.86 (0.073–10)
	Low risk of bias	–	0.81 (0.43–1.6); colistin 31.4%	1 (0.25–3.9); amikacin 50.3%	–	–	1.2 (0.27–5.5)
	Observational studies	0.34 (0.11–0.9); tobramycin 95.7%	0.83 (0.58–1.1); colistin 3.9%	–	0.41 (0.12–1.2)	–	–
	NOS = 9	0.34 (0.094–0.96); tobramycin 95.1%	0.86 (0.54–1.3); colistin 4.2%	–	0.39 (0.1–1.2)	–	–
	NOS < 9	–	0.67 (0.35–1.3); colistin 90.2%	–	–	–	–
	Administration strategy						
	Adjunctive strategy	0.34 (0.14–0.72); tobramycin 95%	0.85 (0.66–1.1); colistin 0.83%	0.8 (0.44–1.5); amikacin 4.1%	0.4 (0.16–0.88)	0.42 (0.14–1.1)	1.1 (0.54–2)
	Substitution strategy	–	–	0.99 (0.088–9.5); amikacin 48.7%	–	–	–

*MDR* multidrug resistance, *NOS* Newcastle-Ottawa scale, *VAP* ventilator-associated pneumonia

**Fig. 3:**
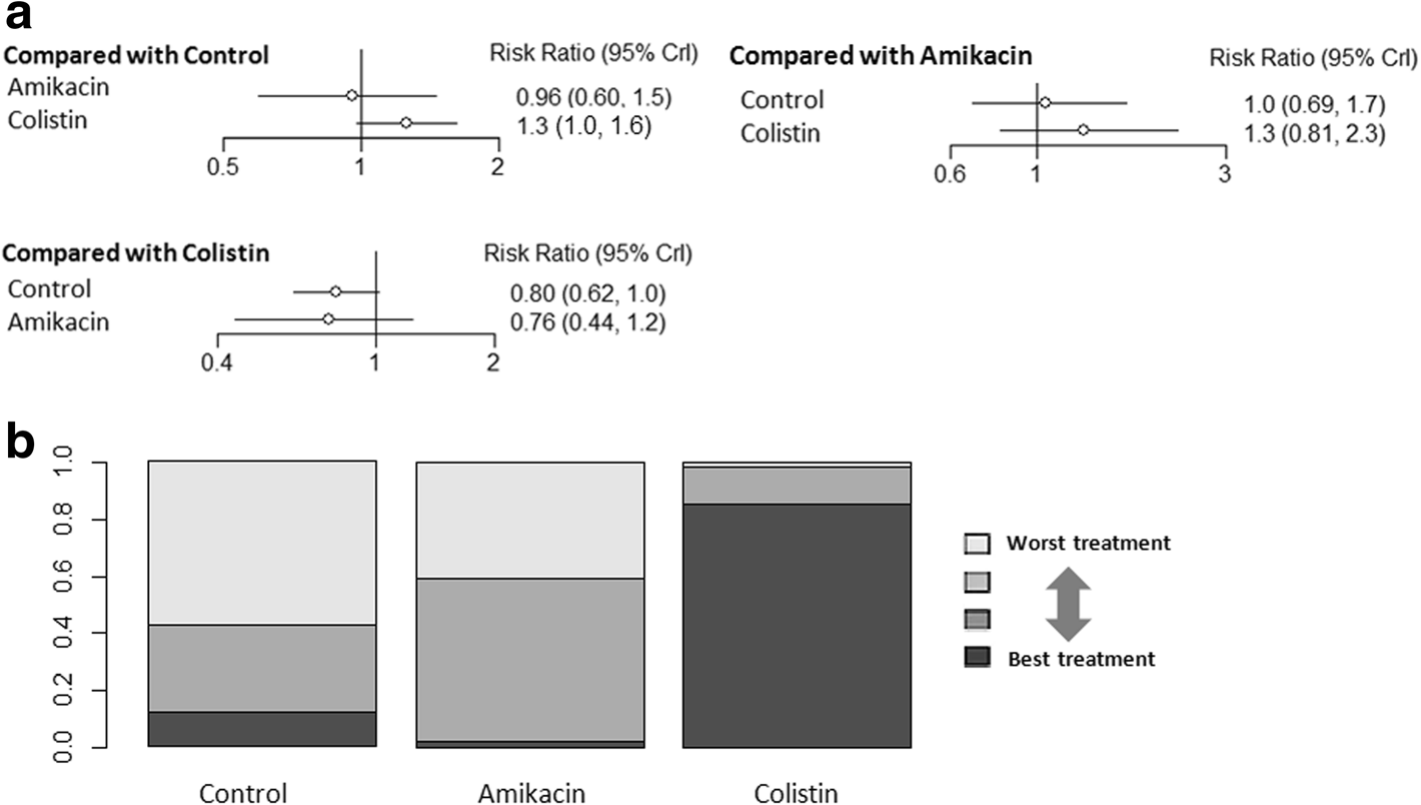
**a** Network estimates among aerosolized antibiotics for microbiological eradication. **b** Rank probabilities among aerosolized antibiotics for microbiological eradication based on the network meta-analysis. The number of patients in each antibiotic arm: control, 271; amikacin, 74; and colistin, 241. CI confidence interval

None of the studies used aerosolized tobramycin to evaluate the effects on microbiological eradication. Aerosolized colistin had a higher rate of microbiological eradication when compared with intravenous antibiotics (NMA: RR 1.3, 95% CI 1.0–1.6; Fig. 3a). There were no significant differences among the other comparisons of the network meta-analysis for microbiological eradication (Fig. 3a). Ranking analysis revealed that colistin presented the highest rate of microbiological eradication (86.2% probability) (Fig. 3b).

**Fig. 4:**
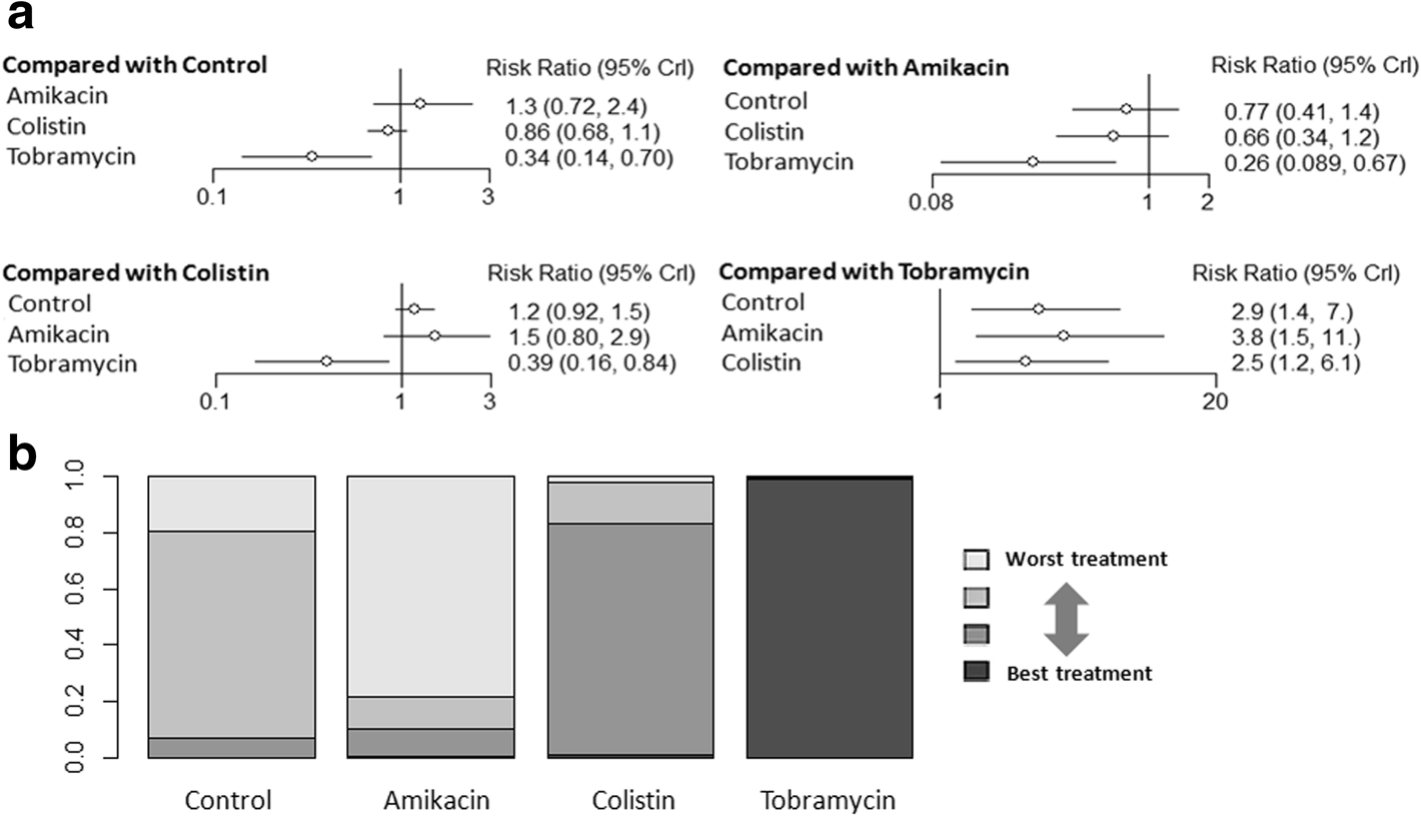
**a** Network estimates among aerosolized antibiotics for mortality. **b** Rank probabilities among aerosolized antibiotics for mortality based on the network meta-analysis. The number of patients in each antibiotic arm: control, 464; amikacin, 138; colistin, 362; and tobramycin, 43. CI confidence interval

Taking intravenous antibiotics as the reference revealed no statistically significant differences in mortality in aerosolized amikacin and colistin, but aerosolized tobramycin showed lower mortality risk (NMA: RR 0.34, 95% CI 0.14–0.70) (Fig. 4a). Aerosolized tobramycin was also associated with significantly lower mortality compared with aerosolized amikacin (NMA: RR 0.26, 95% CI 0.089–0.67) and aerosolized colistin (NMA: RR 0.39, 95% CI 0.16–0.84) (Fig. 4a). Ranking analysis revealed that the hierarchy for efficacy in avoiding death (highest to lowest rank) was tobramycin (98.5% probability), followed by colistin (0.99% probability) and then amikacin (0.37% probability) (Fig. 4b). Table 2 shows the rank probabilities of subgroup analyses that provided the hierarchies for the mortality of the aerosolized antibiotics. Ranking analysis revealed that tobramycin was the best treatment in terms of reducing hospital mortality.

**Fig. 5:**
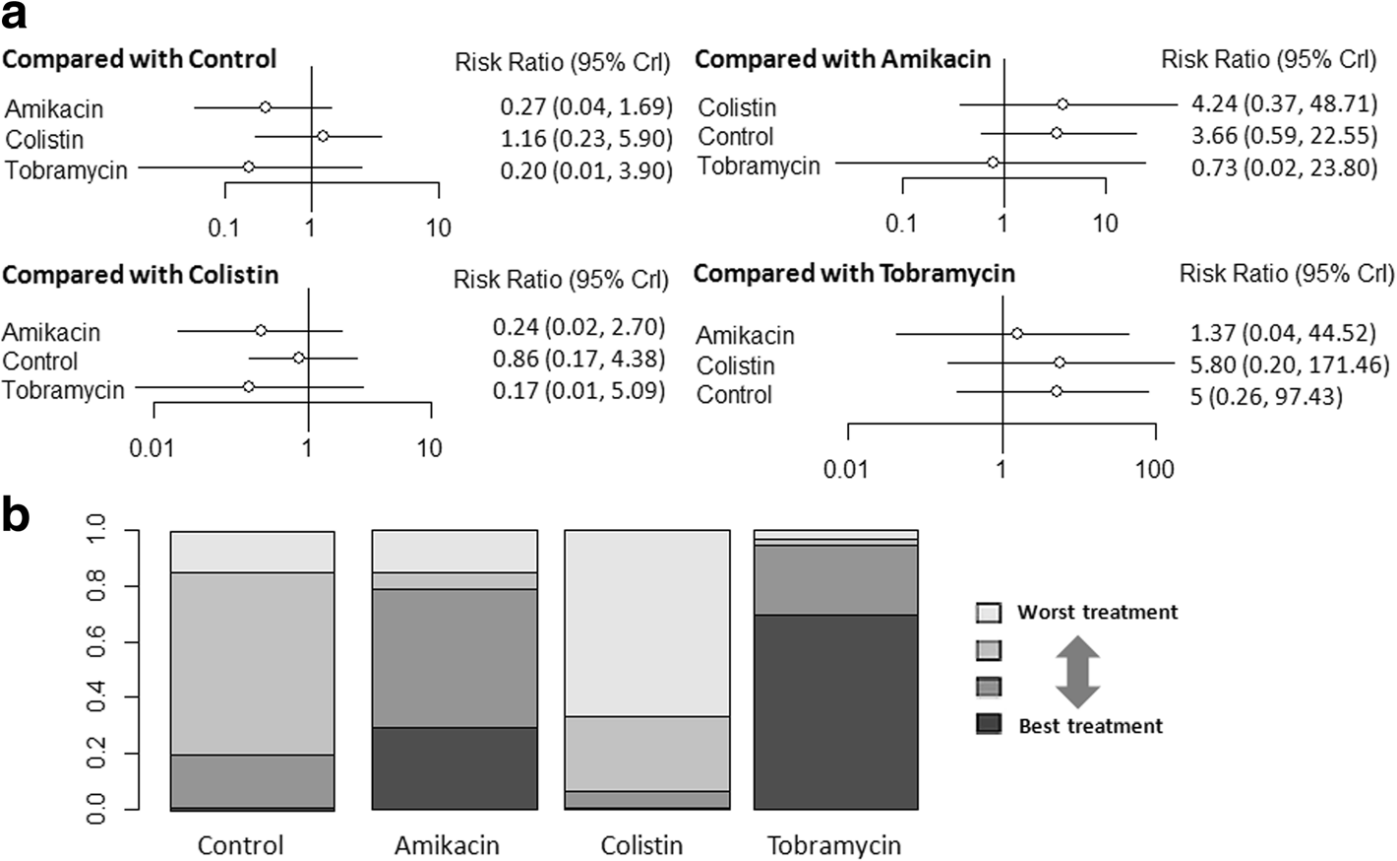
**a** Network estimates among aerosolized antibiotics for nephrotoxicity. **b** Rank probabilities among aerosolized antibiotics for nephrotoxicity based on the network meta-analysis. The number of patients in each antibiotic arm: control, 231; amikacin, 45; colistin, 5; and tobramycin, 230. CI confidence interval

The forest plot for the risk of nephrotoxicity is shown in Fig. 5a. There were no significant differences among any comparisons of the network meta-analysis (Fig. 5a). Ranking analysis revealed that the hierarchy for safety in avoiding renal toxicity (highest to lowest rank) was tobramycin (69.4% probability), followed by amikacin (29.4% probability) and then colistin (0.5% probability) (Fig. 5b).

## Discussion

Previous studies have demonstrated that aerosolized antibiotics affected the clinical prognosis in VAP treatment. Whether aerosolized antibiotics are beneficial or harmful for critically ill patients has long been a matter of debate. In this meta-analysis, we found that aerosolized antibiotics could improve clinical recovery and microbiological eradication. In addition, there were no differences in terms of outcomes such as mortality or nephrotoxicity. The present network meta-analysis and ranking analysis suggest that the probability of being the best aerosolized therapy for VAP was tobramycin with respect to clinical recovery and mortality, and colistin for microbiological eradication. Although this was a hypothesis-generating study, the current study is the first network meta-analysis to compare different aerosolized antibiotics in the treatment of VAP. The present meta-analysis is different from the two previous ones [4, 5]. We have updated the studies for this analysis, separately compared each aerosolized antibiotic with intravenous therapy, assessed the quality of evidence, detected publication bias, and conducted sensitivity analyses. A previous meta-analysis by Zampieri et al. investigated the role of aerosolized antibiotics in VAP treatment [4]. This analysis found the same benefits as our analysis on clinical recovery, mortality, and nephrotoxicity, but aerosolized antibiotics did not increase the rate for microbiological eradication. Contrary to their microbiological eradication findings, our analysis indicated that aerosolized antibiotics also have a beneficial effect. Thus, there may have been an inadequate sample size to correct the type II error in the study of Zampieri et al. In addition, we used network meta-analysis to identify an optimal aerosolized antibiotic for VAP.

VAP remains an important infectious complication of mechanical ventilation and is commonly due to MDR pathogens (*Enterococcus faecium*, *Staphylococcus aureus*, *Klebsiella pneumoniae*, *Acinetobacter baumannii*, *P. aeruginosa*, and enterobacter spp.). Because of MDR pathogens, the capability of commonly used intravenous antibiotics to cross the lung parenchyma is significantly inhibited [26, 27]. Inadequate concentrations of intravenous antibiotics may result in failure to reach inhibitory concentrations. Aerosolized antibiotics may be a useful treatment since aerosolization directly targets the airways and localizes the drug to the pulmonary parenchyma, thus bypassing the poor lung penetration of many antibiotics. Aerosolized antibiotics therefore make it possible to achieve high local antibiotic concentrations relative to an organism’s minimum inhibitory concentration while concurrently minimizing systemic toxicities by concentrating antibiotics in the lungs rather than spreading them throughout the body [28].

The appropriate aerosolized antibiotic remains a matter of controversy, and the most studied are colistin and aminoglycosides. The selection of the aerosolized antibiotic should encompass several properties and characteristics to achieve maximum effectiveness including: 1) activity against the causative pathogen; 2) physical properties to ensure maximal pulmonary delivery and minimal extrapulmonary loss; and 3) the achievement of adequate concentrations in the lung well above the pathogen’s minimum inhibitory concentration, taking into account the need for the prevention of resistance and the presence of biofilm [29].

In the presence of severe experimental lung infection, aminoglycoside plasma concentrations were found in the same range after nebulization and intravenous administration [30]. Furthermore, inhaled aminoglycosides prevent growth of bacterial biofilms [31]. Inhaled tobramycin has a wide range of activity against Gram-negative organisms, including *P. aeruginosa*. Tobramycin is also preferable for inhaled use because of moderately lipophilic and positively charged small molecules [27]. Amikacin is another aminoglycoside. The low bioavailability of nebulized amikacin may result in administration of high dosesand hence potentially increase systemic toxicities. Mohr et al. reported that pharmacokinetic properties and dosing strategies were better defined for inhaled tobramycin than for inhaled amikacin [32]. Studies in animals have shown that aerosolized colistin has a limited systemic diffusion in pneumonia [33]. Likewise, inhaled colistin also has high mucin binding that might lead to insufficient antibiotic effectiveness to kill bacteria [34]. As a consequence, a dosage exceeding the common dose of colistin treating VAP may enhance the rate of clinical recovery and microbiological eradication but increase the risk of toxicity. Therefore, tobramycin may have more potential effects on clinical recovery and be promising for its effects on mortality and nephrotoxicity.

In seeking to optimize treatment, network meta-analysis was used to gain further insight into the best preferred agent. The present network meta-analysis found that aerosolized tobramycin and colistin (especially tobramycin) were more likely to increase the rate of clinical recovery. Aerosolized tobramycin also had additional benefits on mortality, and aerosolized colistin had a higher rate of microbiological eradication. Amikacin had no significant differences in clinical recovery, microbiological eradication, mortality, or nephrotoxicity compared with intravenous antibiotics. Accordingly, the present network meta-analysis and the clustered ranking plot suggest that tobramycin present the best outcome for clinical recovery and mortality, and colistin present the best outcome for microbiological eradication.

Aerosolized antibiotics seem to be attractive alternatives for VAP treatment. However, an in-vivo randomized controlled trial examining adjuvant therapy with aerosolized antibiotics in VAP with varying degrees of bacterial resistance has shown that aerosolized antibiotics have no effect on the clinical course, including the serial Clinical Pulmonary Infection Score (CPIS), clinical cure rates, ventilator-free and ICU days, or mortality [24]. Despite the weak evidence in our analysis suggesting that the administration of aerosolized antibiotics such as colistin or tobramycin instead of the administration of intravenous antibiotics might be a good option for the treatment of VAP, there may as yet be no recommendations for using them in standard clinical practice. The rationale for the recommendation of aerosolized antibiotics requires other large multicenter trials to determine if these preliminary findings will result in better clinical activity and decreased microbial resistance in patients with VAP.

Some important limitations of the study should not be overlooked. First, different criteria for clinical recovery, microbiological eradication, and nephrotoxicity in the included trials might affect the robustness of our findings. The endpoint of microbiologic eradication is of uncertain value when measured during the use of inhaled antibiotics since the inhaled antibiotics are in the respiratory sample sent for culture which may influence the accuracy of the endpoint. Second, there are many factors which may impact the effect of inhaled antibiotics including types of nebulizers, ventilator settings, ventilator and circuit connections, and circuit humidification and filtering. We could not obtain sufficient data suggesting that other study design issues should be addressed. Third, the eligible studies had substantial differences in terms of the causative microorganisms, and the impact of inhaled antibiotics might vary against different pathogens. Fourth, a major concern when small studies are included in our analysis is the presence of a “small-study effect”, which arises from publication bias and selection bias and results in lower methodological quality [35]. The small-study effect was present in the analysis of treatment success in VAP patients with respect to aerosolized antibiotics. This should be taken into careful consideration when evaluating the results of the present analysis. Fifth, although network meta-analysis allowed us to compare the efficacy and safety of inhaled antibiotics, we acknowledge the limitation that interpretation of a network meta-analysis relies on the insufficient indirect comparison of outcomes through common comparators. Sixth, we included observational studies and randomized controlled trials; however, observational studies have the risk of overrated pooled estimates.

## Conclusions

Aerosolized antibiotics appear to be useful treatment for VAP with respect to clinical recovery and microbiological eradication, and do not seem to increase mortality or nephrotoxicity risks. Our network meta-analysis in patients with VAP suggests that clinical recovery benefits are associated with aerosolized tobramycin and colistin (especially tobramycin), microbiological eradication with aerosolized colistin, and survival with aerosolized tobramycin, mostly based on observational studies. Moreover, we should take into account that some studies using aerosolized antibiotics failed to show an improvement, and data of other studies are still not published. Due to the low level of evidence, definitive recommendations cannot be made before additional large randomized studies are carried out.

## Key messages

- Aerosolized antibiotics appear to be beneficial for the treatment of VAP.
- The current evidence from network meta-analysis indicates that aerosolized tobramycin is associated with the best outcome on clinical recovery and mortality.
- The current evidence from network meta-analysis indicates that aerosolized colistin is associated with improved clinical recovery and microbiological eradication.

## Additional files

Additional file 1: **Figure S1.** Forest plots showing the effect of aerosolized antibiotics on (A) clinical recovery, (B) microbiological eradication, (C) mortality, and (D) nephrotoxicity. (TIF 8222 kb)

Additional file 2: **Figure S2.** Funnel plots for the rate of (A) clinical recovery, (B) microbiological eradication, (C) mortality, and (D) nephrotoxicity. (TIF 2106 kb)

Additional file 3: **Figure S3.** Sensitivity analyses of the included studies reporting aerosolized antibiotics on (A) clinical recovery, (B) microbiological eradication, (C) mortality, and (D) nephrotoxicity. (TIF 4998 kb)

## Abbreviations

CI: Confidence interval
CI: Confidence interval
HAP: Hospital-associated pneumonia
HAP: Hospital-associated pneumonia
MDR: Multidrug resistant
NMA: Network meta-analysis
NMA: Network meta-analysis
NOS: Newcastle-Ottawa scale
NOS: Newcastle-Ottawa scale
RCT: Randomized controlled trials
RR: Risk ratio
RR: Risk ratio
VAP: Ventilator-associated pneumonia
VAP: Ventilator-associated pneumonia
