# Supplementary figures and images for: Aerosolized antibiotics for ventilator-associated pneumonia: a pairwise and Bayesian network meta-analysis
Source: Crit Care. 2018 Nov 15;22:301. doi: 10.1186/s13054-018-2106-x (PMC6238320; doi:10.1186/s13054-018-2106-x)

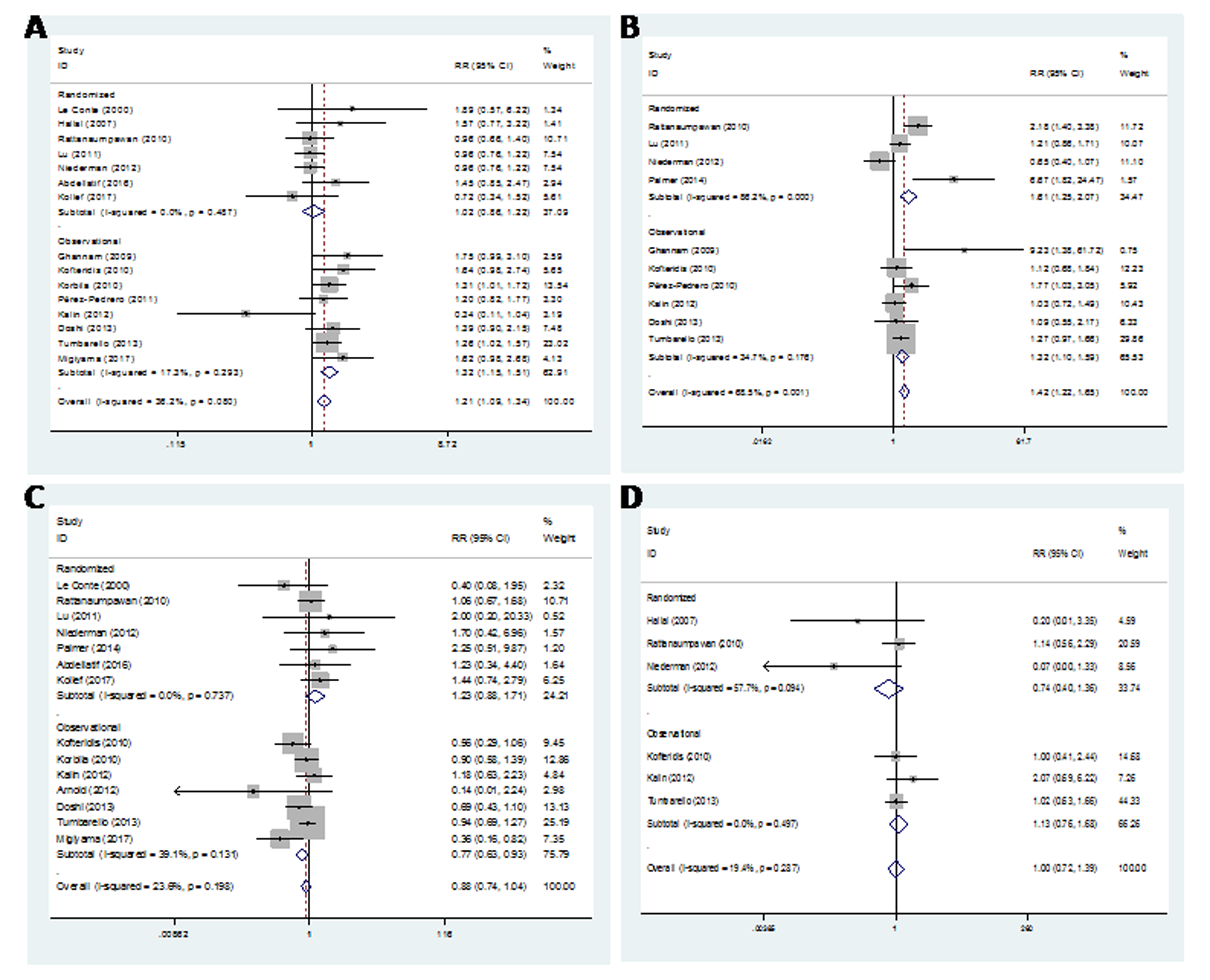

Supplement: Supplementary file 1 — Figure S1. Forest plots showing the effect of aerosolized antibiotics on (A) clinical recovery, (B) microbiological eradication, (C) mortality, and (D) nephrotoxicity. (TIF 8222 kb) [file 13054_2018_2106_MOESM1_ESM.tif]

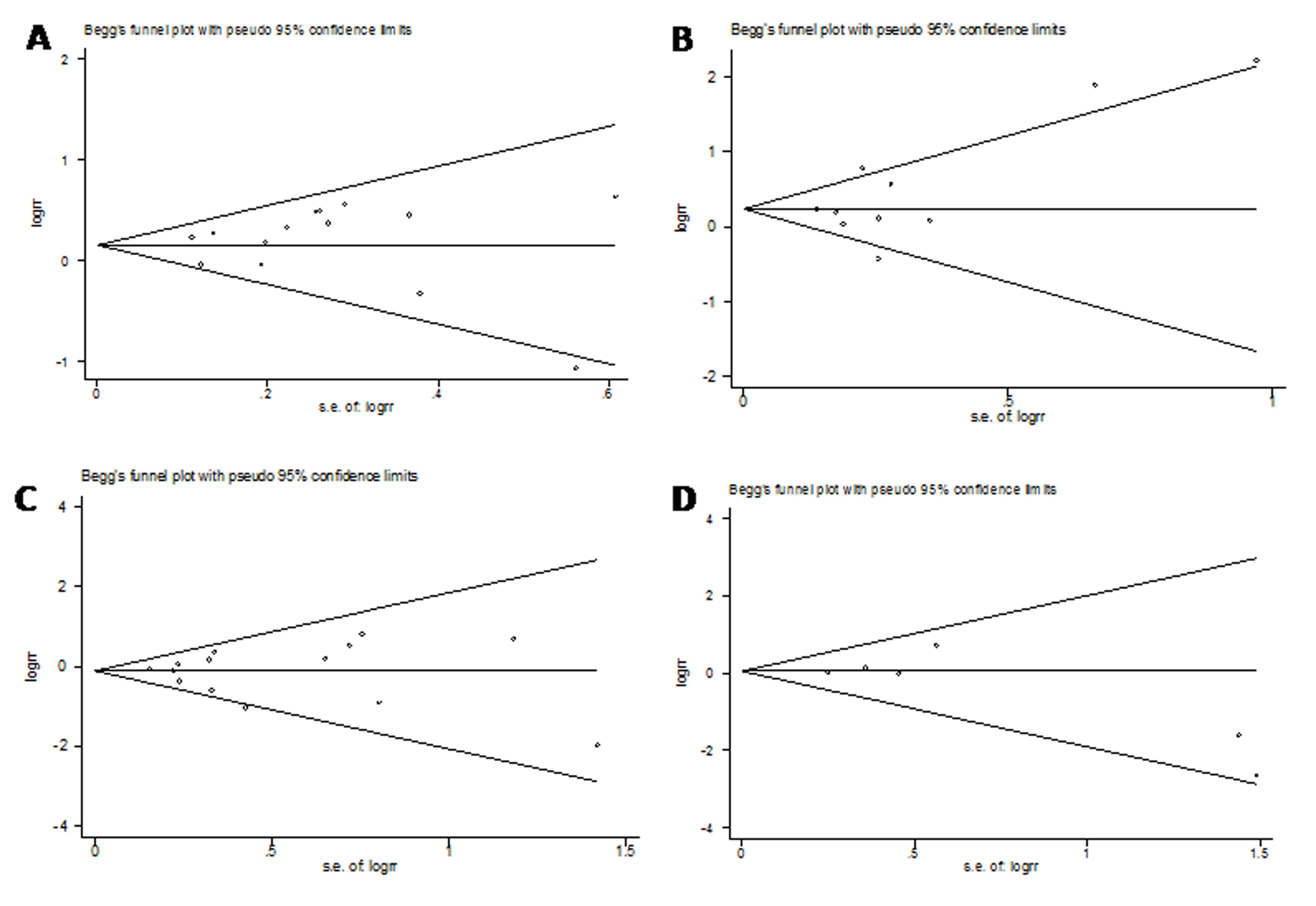

Supplement: Supplementary file 2 — Figure S2. Funnel plots for the rate of (A) clinical recovery, (B) microbiological eradication, (C) mortality, and (D) nephrotoxicity. (TIF 2106 kb) [file 13054_2018_2106_MOESM2_ESM.tif]

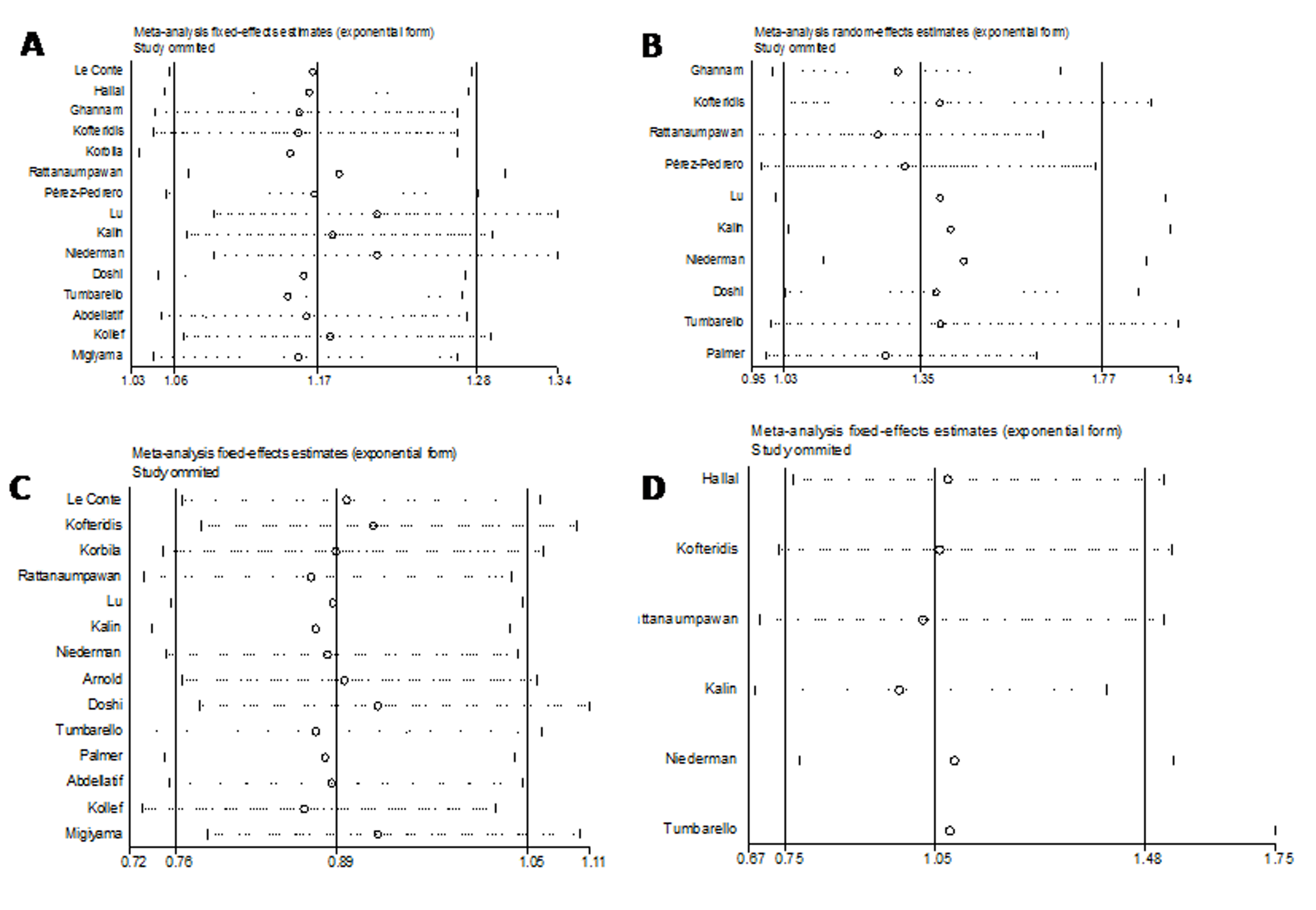

Supplement: Supplementary file 3 — Figure S3. Sensitivity analyses of the included studies reporting aerosolized antibiotics on (A) clinical recovery, (B) microbiological eradication, (C) mortality, and (D) nephrotoxicity. (TIF 4998 kb) [file 13054_2018_2106_MOESM3_ESM.tif]
